# Knowledge, attitude, and practices associated with avian influenza among undergraduate university students of East Java Indonesia: A cross-sectional survey

**DOI:** 10.12688/f1000research.74196.1

**Published:** 2022-01-28

**Authors:** Saifur Rehman, Fedik Abdul Rantam, Khadija Batool, Attaur Rahman, Mustofa Helmi Effendi, Jola Rahmahani, Muhsin Jamal

**Affiliations:** 1 Division of Veterinary Public Health Faculty of Veterinary Medicine, Universitas Airlangga, Surabaya, East Java, 60115, Indonesia; 2 Laboratory of Virology and Immunology Division of Microbiology, Universitas Airlangga, Surabaya, East Java, 60115, Indonesia; 3 Medicine, Service Institute of Medical Sciences Lahore Pakistan, Surabaya, Islamic, 40050, Pakistan; 4 College of Animal Husbandry and Veterinary Sciences, Abdul Wali Khan University Mardan, Mardan, 23200, Pakistan; 5 Department of Microbiology, Abdul Wali Khan University, Mardan, Pakistan

**Keywords:** Avian Influenza, Knowledge, Attitude, Practices, Public Health, Undergraduates

## Abstract

**Background:** Several public health strategic actions are required for effective avian influenza (AI) prevention and control, as well as the development of a communication plan to keep undergraduate students sufficiently informed on how to avoid or reduce exposure. The aim of the survey was to measure the level of knowledge, attitudes and practices (KAPs) toward AI among undergraduate university students in East Java, Indonesia, and observe the correlation between KAPs and the factors associated with the control and prevention of AI.

** Methods: **A cross-sectional survey was conducted among undergraduate students to collect information about AI-related KAPs. Students were selected from three faculties of Universitas Airlangga Surabaya Indonesia (Faculty of Veterinary Medicine, Faculty of Fisheries and Marine, and Faculty of Science and Technology). Students voluntarily responded to a pre-designed questionnaire.

**Results: **A total of 425 students (222 female; and 203 male), of ages ranging from 18 years (n=240) to 20-30 years (n=185), responded to the survey. This cohort consisted of 157 students from the Faculty of Fisheries and Marine, 149 from the Faculty of Veterinary Medicine, and 119 from the Faculty of Science and Technology. The results indicated that appropriate knowledge was obtained by 76.94% of students; significantly higher levels were seen in Faculty of Veterinary Medicine students as compared to the other two faculties (p<0.05). 72.89% of students documented positive attitudes; veterinary medicine students had significantly more positive attitudes than other faculties (p<0.05). Proactive behaviors were observed in 56.90% of students. The aggregate scores for KAPs were 6.93 ± 0.77 (range: 0-9) for knowledge, 7.6 ± 1.25 (range: 0-10) for attitude, and 9.1 ± 1.5 (range: 0-12) for practice.

## Introduction

Avian influenza (AI) is a highly contagious viral zoonotic disease, and has become a serious public health concern in the last two decades as a result of a significant increase in the interspecies transmission of AI viruses from diseased birds to humans ^1, 2^. Since the first laboratory-confirmed case was reported in Hong Kong in 1997, there have been 860 confirmed human infections with the avian H5N1 virus, resulting in 454 deaths worldwide ^1^. To date, only the H5 and H7 AI subtypes have been associated with naturally occurring, highly pathogenic AI A (HPAI) viruses that cause acute clinical disease in chickens, ducks, turkeys, and other economically significant birds. The majority of AI viruses of the subtypes H5 and H7 identified from birds, have been found to be low-pathogenic to poultry. All H5 and H7 viruses have been categorized as Notifiable Avian Influenza (NAI) viruses, because there is a possibility for lentogenic H5 or H7 viruses to become velogenic through mutation. Humans, rats and mice, weasels and ferrets, pigs, cats, tigers, and dogs have all been found to be carriers of the virus ^3^. In various countries, including Vietnam, Indonesia, Thailand, China, Cambodia, and, more recently, Turkey and Iraq, AI strains have been found in birds, including wild and commercial poultry ^4^.

In Indonesia, the majority of human cases of HPAI H5N1 have occurred in the western part of the main Island of Java. Although minimal, non-sustained human-to-human transmission has undoubtedly occurred, and the transmission was linked to poultry exposure, such as direct or close contact with infected or dead birds or visiting a live poultry market ^1, 5– 10^. Late presentation to health care and hospitalization, delayed clinical detection of AI and delayed antiviral therapy have all been related to increased mortality from HPAI H5N1 virus infection in Indonesia ^7, 11– 13^. Knowledge, attitude, and practices (KAPs) are one of the most important factors in controlling and preventing the spread of certain diseases or infections to public health ^14– 16^. In the past few years, infected countries like Thailand, China, Italy, Turkey, and Afghanistan have been studying KAPs towards AI. Workers in the poultry industry had the highest risk of contracting AI. A study of Italian poultry workers was undertaken to assess their KAPs toward AI, and it was discovered that workers' awareness of transmission and prevention measures might be enhanced because of their close contact with poultry ^17^. A KAP questionnaire is a comprehensive survey conducted on a specific population to determine a population's knowledge (K), attitudes (A), and practices (P) on a particular topic. In most KAPs studies, an interviewer uses a standardized, structured questionnaire to obtain data orally ^18^. An earlier survey was carried out in Thailand among the rural community on KAPs towards AI, revealing that a public education campaign was beneficial in promoting AI disease prevention methods to the public ^19^. However, there is a paucity of research on the KAPs towards AI in Indonesia. Considering the pandemic classification of AI and its critical consequences in terms of economic losses to the poultry industry and serious deleterious effects on public health, a survey was undertaken among undergraduate university students at a public sector university, to explore the benefits of adopting a KAP strategic model in controlling and preventing AI in Indonesia. The aims of the survey was to measure the level of KAPs towards avian flu among the students, and observe the correlation between KAPs and the factors associated with control and prevention of AI. The findings will significantly help to understand the basic knowledge of AI, its clinical manifestation, pathogenesis, routes of transmission, broad range of hosts, and pandemic nature of the virus, acquired by undergraduate students in particular, and Indonesians in general.

## Methods

The respondents of this survey were the undergraduate students (from the Faculty of Veterinary Medicine, Faculty of Fisheries and Marine, and Faculty of Science and Technology) of the University of Airlangga Surabaya Indonesia. The study was conducted from April 23 to May 20, 2021, during the peak of the COVID-19 pandemic. After obtaining ethical approval from the Faculty of Veterinary Medicine Research Ethics Committee, the respondents were interacted with through face-to-face meetings to respond to the study questionnaire. This measure was taken due to the practical nature of this study that required self-administration for rapid data collection. The advantage of this method includes handling missing values, consistency of data findings, transparency of personal interest and the study framework as per literature guidance ^20^.

We contacted 425 undergraduate students from three different faculties, of Universitas Airlangga of ages ranging between 18 to 30 years to get the questionnaire filled. Informed consent was obtained from participants and anonymity of personal information was also considered. The sample size was determined based on a 5% precision level of faculty population.

These faculties were chosen because most students from the Faculty of Veterinary Medicine carry out research related to zoonotic diseases; while few students from other faculty carry out research on zoonotic diseases, they are part of their curriculum, such as AI, salmonella, and klebsiella. All participants were informed about the survey before filling the questionnaire with the following statements “All participation in this study is completely voluntary, if you decide not to participate there will not be any negative consequences. Please be aware that if you decided to participate, you may stop participating any time and you may decide not to answer any specific question. The aim of the survey is to measure the level of KAPs toward avian flu among the students. You will receive no direct benefits from participating in this research study. However, your responses may help us learn more about KAPs of avian influenza”.

### Scoring and recording of response variables

The questionnaire contained four demographic characteristics (age, gender, hometown, and name of faculty); KAP parameters were a set of nine AI knowledge variables (avian influenza is a contagious infection, avian influenza is caused by highly pathogenic influenza A [H5N1] virus, avian influenza is similar to swine influenza regarding their signs and symptoms [transmission] animal-to-animal, animal-to-human, human-to-human (risk group) poultry workers, butchers, veterinarians); five AI attitude variables (washing hands before eating and after touching raw poultry meat, using gloves to touch raw poultry meat, preparing raw poultry and other foods using different knives, and cleaning the cutting boards after preparing raw poultry meat); and six AI practice variables (washing hands with soap before and after eating, covering nose when sneezing and coughing, wearing a surgical mask and consultation with the doctor promptly in case of suspected contamination). All questions were developed based on a published questionnaire from a study on Italian poultry workers ^21^. For this KAP questionnaire, a standard scoring method was used encompassing all KAP sections. In the knowledge section, correct answers were scored 1 point and 0 points were given for incorrect responses, while in the attitude section positive options obtained 2 points, neutral options obtained one point, while negative options get zero points. Similarly, in the practice portion, 2 points were awarded for proactive actions, 1 point was given for neutral actions, and 0 points for passive actions.

### Data processing and analysis

The data collected through a standard KAP questionnaire module were subjected to statistical analysis by using the SPSS 25.0 software package. Both descriptive and inferential statistical tests (Chi-square) were applied to compare categorical response variables and ratios, and to assess their statistical significance (p<0.05).

## Results

### Respondent demographics

All undergraduate students (n=425) were selected from three different faculties of Universitas Airlangga Surabaya Indonesia. Among them, 52.2% (222 out of 425) were females and 47.8% (203 out of 425) were males. A total of 35.05% (149 out of 425) of the respondents were from the Faculty of Veterinay Medicine, 36.94% (157 out of 425) from the Faculty of Fisheries and Marine, and 28% (119 out of 425) students from the Faculty of Science and Technology. A 56.5% fraction (240 out of 425) of participants were <20 years old while 43.5% (185 out of 43.5%) were aged between 20–30 years.

### Knowledge of AI

AI-associated knowledge was assessed by five questions. Each question-and-answer is described with graded scores in Table 1. Among the total 3,825 answers, 2,943 (76.94%) were correct. Significantly higher scores were found in Faculty of Veterinary Medicine students for K1, K5, K6, and K8 as compared to those from the Faculty of Fisheries and Marine and the Faculty of Science and Technology (p<0.05), but no other statistical significance was found among the groups ( Table 2).

**Table 1.** Avian influenza knowledge among undergraduate students.

Instrument question	Options	Determination/ score	N%
Definition			
K1: Avian influenza is a contagious infection	True	Correct/ 1	409 (96.2)
	False	Incorrect/ 0	4 (0.9)
	Do not know	Incorrect/ 0	12 (2.8)
K2: It is caused by the Highly Pathogenic Influenza A (H5N1) virus	True	Correct/ 1	382 (89.9)
	False	Incorrect/ 0	10 (2.4)
	Do not know	Incorrect/ 0	33 (7.8)
K3: Avian influenza is similar to swine influenza regarding its signs and symptoms	True	Correct/ 1	255 (60)
	False	Incorrect/ 0	37 (8.3)
	Do not know	Incorrect/ 0	133 (31.3)
Mode of transmission			
K4: Animal-to-animal	True	Correct/ 1	332 (78.1)
	False	Incorrect/ 0	59 (13.9)
	Do not know	Incorrect/ 0	34 (8)
K5: Animal-to-human	True	Correct/ 1	347 (81.6)
	False	Incorrect/ 0	50 (11.8)
	Do not know	Incorrect/ 0	28 (6.6)
K6: Human-to-human	True	Correct/ 1	238 (56)
	False	Incorrect/ 0	120 (28.2)
	Do not know	Incorrect/ 0	67 (15.8)
Risk groups			
K7: Butchers	True	Correct/ 1	304 (71.5)
	False	Incorrect/ 0	52 (12.2)
	Do not know	Incorrect/ 0	69 (16.2)
K8: Poultry workers	True	Correct/ 1	367 (86.4)
	False	Incorrect/ 0	32 (7.5)
	Do not know	Incorrect/ 0	26 (6.1)
K9: Veterinarians	True	Correct/ 1	309 (72.7)
	False	Incorrect/ 0	35 (8.2)
	Do not know	Incorrect/ 0	81 (19.1)

**Table 2.** Comparing knowledge of avian influenza among different faculties.

No of Instrument	Faculty of Veterinary Medicine (n=149)				Faculty of Fisheries and Marine (n=157)				Faculty of Science and Technology (n=119)			
	Male % (n=104)	Female % (n=45)	X ^2^	P-value	Male % (n=67)	Female % (n=90)	X ^2^	P-value	Male % (n=32)	Female% (n=87)	X ^2^	P-value
K1 correct	100 (96.2)	43 (95.6)	6.354	0.042	64 (95.5)	86 (95.6)	3.942	0.139	31 (96.9)	82 (94.3)	2.031	0.362
K2 correct	98 (94.2)	40 (88.9)	1.311	0.252	61 (91)	83 (92.2)	0.084	0.959	32 (100)	81 (93.1)	2.324	0.127
K3 correct	89 (85.6)	38 (84.4)	2.380	0.304	57 (85.1)	76 (84.4)	0.148	0.929	29 (90.6)	69 (79.3)	2.574	0.276
K4 correct	97 (93.3)	44 (97.8)	2.245	0.325	64 (95.5)	82 (91.1)	1.754	0.416	32 (100)	79 (90.8)	3.155	0.207
K5 correct	93 (89.4)	36 (80)	8.330	0.016	57 (85.1)	79 (87.8)	0.286	0.867	29 (90.6)	80 (92)	0.562	0.755
K6 correct	82 (78.9)	18 (40)	22.21	<0.001	50 (74.6)	55 (61.1)	4.323	0.115	23 (72)	56 (64.4)	0.995	0.608
K7 correct	98 (94.2)	40 (88.9)	1.311	0.252	61 (91)	85 (94.4)	0.409	0.681	31 (96.9)	78 (89.7)	1.584	0.208
K8 correct	96 (92.3)	31 (68.9)	14.46	0.001	54 (80.6)	80 (88.9)	2.325	0.313	28 (87.5)	75 (86.2)	3.294	0.193
K9 correct	95 (91.3)	37 (82.2)	3.251	0.197	60 (89.6)	80 (88.9)	0.985	0.611	29 (90.6)	74 (85.1)	1.283	0.526

Table 2 lists the questions and correct answers for each variable. The percentage of correct information between different groups was compared using the Chi-square test.

### Attitude towards AI

There were five categories of AI attitude questions. Each question-and-answer option is presented in detail with scores in Table 3. In total, 1,549 (72.89 %) of the 2,125 responses showed a positive attitude ( Table 3). Faculty of Veterinary Medicine students' scores were significantly higher than other faculties for A4 and A5 (p<0.05), while no other variable showed statistically significant differences between faculties ( Table 4).

**Table 3.** Undergraduate students' attitudes toward avian influenza.

Study instruments	Options	Determination/ score	N%
We should wash our hands with soap			
A1: Before eating	Strongly agree	Positive/ 2	379 (89.2)
	Agree	Neutral/ 0	45 (10.6)
	Uncertain	Negative/ 1	1 (0.2)
A2: After touching raw poultry meat	Strongly agree	Positive/ 2	312 (73.4)
	Agree	Neutral/ 0	89 (21)
	Uncertain	Negative/ 1	24 (5.6)
A3: Using gloves to touch raw poultry meat is a good hygienic practice	Strongly agree	Positive/ 2	277 (65.2)
	Agree	Neutral/ 0	104 (24.5)
	Uncertain	Negative/ 1	44 (10.3)
A4: Preparing raw poultry and other foods using different knives is a good practice	Strongly agree	Positive/ 2	275 (64.7)
	Agree	Neutral/ 0	166 (27.3)
	Uncertain	Negative/ 1	34 (8)
A5: We should clean the cutting boards after preparing raw poultry meat	Strongly agree	Positive/ 2	306 (72)
	Agree	Neutral/ 0	99 (23.3)
	Uncertain	Negative/ 1	20 (4.7)

**Table 4.** Comparison of attitudes of different faculties toward avian influenza.

No of Instruments	Faculty of Veterinary Medicine (n=149)				Faculty of Fisheries and Marine (n=157)				Faculty of Science and Technology (n=119)			
	Male % (n=104)	Female % (n=45)	X ^2^	P-value	Male % (n=67)	Female % (n=90)	X ^2^	P-value	Male % (n=32)	Female% (n=87)	X ^2^	P-value
A1 Positive	89 (85.6)	42 (93.3)	5.267	0.072	57 (85.1)	82 (91.1)	2.234	0.327	31 (96.9)	76 (87.4)	2.338	0.126
A2 Positive	81 (77.9)	38 (84.4)	0.841	0.359	52 (70.3)	74 (82.2)	0.515	0.473	28 (87.5)	72 (82.8)	0.392	0.531
A3 Positive	75 (72.1)	30 (66.6)	0.474	0.789	46 (68.7)	65 (72.2)	0.526	0.769	25 (78.1)	62 (71.3)	1.049	0.592
A4 Positive	88 (84.6)	26 (81.2)	18.01	<0.001	52 (70.3)	69 (76.7)	.187	0.911	25 (78.1	69 (79.3)	0.125	0.939
A5 Positive	87 (83.6)	30 (66.6)	7.598	0.022	51 (76.1)	73 (81.1)	2.886	0.236	27 (84.3)	69 (79.3)	1.275	0. 529

Table 4 lists the questions and correct options for each variable. The percentage of positive attitudes between different groups was compared using a Chi-square test.

### Practice related to AI among the respondents

Six questions were used to assess practices related to AI; each question-and-response option, along with their graded scores, are given in Table 5 and Table 6. Out of 2,550 responses, 1,451 (56.90%) had adopted proactive practices. For questions P1, P4, and P5, veterinary students scored significantly higher than Faculty of Fisheries and Marine students. Students from the Faculty of Science and Technology scored much higher on question P4 than Faculty of Fisheries and Marine students (p<0.05) ( Table 6).

**Table 5.** Practices toward avian influenza among undergraduate students.

Study instruments	Options	Determination/ score	N%
P1: I wash my hands with soap before eating	All the times	Proactive/ 2	270 (63.5)
	Sometimes	Neutral/ 1	155 (36.5)
	Never	Passive/ 0	-
P2: I wash my hands with soap after eating	All the times	Proactive/ 2	265 (62.4)
	Sometimes	Neutral/ 1	148 (34.8)
	Never	Passive/ 0	12 (2.8)
**I cover my nose and mouth when I am**			
P3: Sneezing	All the times	Proactive/ 2	296 (69.6)
	Sometimes	Neutral/ 1	116 (27.3)
	Never	Passive/ 0	13 (3.1)
P4: Coughing	All the times	Proactive/ 2	273 (64.2)
	Sometimes	Neutral/ 1	137 (32.2)
	Never	Passive/ 0	15(3.5)
**When I have influenza-like symptoms such as cough, runny nose, and** ** sore throat**			
P5: I wear a surgical mask	All the times	Proactive/ 2	216 (50.8)
	Sometimes	Neutral/ 1	183 (43.1)
	Never	Passive/ 0	26 (6.1)
P6: I consult the doctor promptly	All the times	Proactive/ 2	131 (30.8)
	Sometimes	Neutral/ 1	241 (56.7)
	Never	Passive/ 0	53 (12.5)

**Table 6.** Comparison of avian influenza practices between different faculties.

No of Variables	Faculty of Veterinary Medicine (n=149)				Faculty of Fisheries and Marine (n=157)				Faculty of Science and Technology (n=119)			
	Male % (n=104)	Female % (n=45)	X2	P -value	Male % (n=67)	Female % (n=90)	X2	P-value	Male % (n=32)	Female% (n=87)	X2	P- value
P1 Proactive	49 (47.1)	35 (77.8)	12.007	0.001	41 (61.2)	49 (54.4)	0.715	0.398	16 (50)	49 (56.3)	0.377	0.539
P2 Proactive	57 (54.8)	31 (68.9)	4.556	0.102	40 (60)	53 (58.9)	0.606	0.739	21 (65.6)	51 (58.6)	1.062	0.588
P3 Proactive	61 (58.7)	28 (62.2)	4.556	0.102	38 (56.7)	56 (62.2)	0.879	0.644	22 (68.8)	52 (60)	0.911	0.634
P4 Proactive	58 (55.7)	30 (66.6)	7.820	0.020	37 (41.1)	56 (55.2)	0.816	0.665	25 (78.1)	47 (54.02)	6.318	0.042
P5 Proactive	51 (49)	28 (62.2)	5.851	0.054	37 (41.1)	45 (50)	0.892	0.640	23 (71.9)	46 (52.9)	4.809	0. 090
P6 Proactive	41 (39.4)	18 (40)	1.679	0.432	28 (41.8)	32 (35.6)	1.128	0.569	18 (56.3)	35 (40.2)	2.538	0. 281

Table 6 lists the questions and proactive options for each variable. The percentage of proactive options in different groups was compared using the Chi-square test.

## Discussion

The goal of the study was to gather data on AI-related KAPs among undergraduate students from three different faculties of Universitas Airlangga, Indonesia. We found that all the participants were aware of AI. Additionally, public health education was identified as a useful method for preventing and controlling public health emergencies, as well as improving public preparedness in the case of any pandemic ^22^. It encourages the public to gain adequate knowledge to reduce stress and anxiety, develop a positive attitude and keep desirable behaviors under the situation of pandemic ^23^. All of these KAP components have been deemed essential for efficient pandemic prevention and control. This cross-sectional study of 425 undergraduate students indicates that the majority of them were well-informed about AI knowledge, had a positive attitude, and engaged in proactive practices, showing that major public education efforts offered effective health awareness benefits. This finding reflects several previous studies reports on H1N1-related KAPs among university students in South Korea, the United Kingdom (UK), and Hong Kong ^24– 26^. Our study revealed that Veterinary Medicine students scored much higher on knowledge than students from other faculties, which could be explained by their exposure to, and training in, clinical medicine and veterinary public health, the concept of One Health and zoonosis. Their obligations and responsibilities as future public veterinary health experts to combat any pandemic are assumed to motivate them to adopt more positive attitudes and proactive behaviors in the event of a public health emergency ^27^. In the attitude section, students from the Faculty of Veterinary Medicine showed a significantly greater positive attitude than the other two faculties, indicating that veterinary students were more aware of the zoonotic importance of AI. This could explain the importance of veterinary education in the current study regarding One Health approaches and the role of the veterinarian in an eco-friendly environment ^28^. Our findings are compatible with the results of previous studies on KAPs towards H1N1 among university students of Hong Kong, South Korea, and the UK ^24– 26^.

In the practice section, students from the Faculty of Veterinary Medicine and Faculty of Science and Technology showed significantly higher scores (p<0.05) as compared to the Faculty of Fisheries and Marine students. These standard practices noted in the current study showed a positive correlation between science and technology and veterinary science courses with education on infectious diseases like AI. Similarly, a prior COVID-19-related KAP study are conducted among undergraduate students in China, revealed that medical and health science students have more proactive practices as compared to other students from different fields of education ^29^.

There are some limitations to our research that must be noted. First, the nature of the cross-sectional study design limits the ability to draw causal inferences from the observed relationships. Second, our participants were recruited from three faculties within a single university, and attended the university during a pandemic for their research activity, while the majority of students stayed at home at the time of the survey due to the COVID-19 pandemic lockdown.

To our knowledge, this is the first study of current KAPs related to AI among Indonesian undergraduate students at any university, and it provides useful information regarding public health education and preventative measures in Indonesian universities during any pandemic. Our findings revealed that most of the undergraduate students at the University of Airlangga have a baseline knowledge of AI, although their scores may vary depending on the Faculty. Attitude towards AI showed a discrepancy among the Faculty students. Overall, our findings showed that faculties other than Veterinary Medicine have an impact on students' reactions to AI-related KAPs. In the educational and health sectors, public health education and awareness initiatives are conducted regarding infectious diseases.

## Conclusions

According to the findings of this study, the majority of undergraduate students grasped the basic information, had a positive attitude, and showed a proactive behavior toward AI, demonstrating the efficacy and success of current public health education initiatives. However, health and educational institutions should adopt public health trainings, prepare the global population and strengthen their prophylactic measures against any pandemic. The results suggest that the students from the Faculty of Fisheries and the Faculty of Science and Technology should be taken into consideration for future strategic studies of awareness campaigns, public health concerns preparedness, and proactiveness in case of any pandemic.

## Data availability

### Underlying data

Figshare: Knowledge, attitude and practices associated with Avian influenza among undergraduate university students of East Java Indonesia: A cross-sectional survey, https://doi.org/10.6084/m9.figshare.16664488.v1 ^30^

This project contains the following underlying data:

- Final for spss.xlsx (survey answers data)
- Spss Survey final File.sav

### Extended data

Figshare: Knowledge, attitude and practices associated with Avian influenza among undergraduate university students of East Java Indonesia: A cross-sectional survey, https://doi.org/10.6084/m9.figshare.16664488.v1 ^30^

This project contains the following extended data:

- Questionnaire.docx
- Tables.docx

Data are available under the terms of the Creative Commons Zero "No rights reserved" data waiver (CC0 1.0 Public domain dedication). 
