# Supplementary material for: Knowledge, attitude, and practices associated with avian influenza among undergraduate university students of East Java Indonesia: A cross-sectional survey
Source: F1000Res. 2022 Jan 28;11:115. [Version 1] doi: 10.12688/f1000research.74196.1 (PMC8921684; doi:10.12688/f1000research.74196.1)
Supplement: Peer review history

# Peer review: Reviewer response for version 1

**DOI:** 10.5256/f1000research.77920.r123902

**Published:** 2022-03-14

**Authors:** Farazi Muhammad Yasir Hasib

**Affiliations:** 1 Department of Pathology and Parasitology, Faculty of Veterinary Medicine, Chattogram Veterinary and Animal Sciences University, Chattogram, Bangladesh; 2 Infectious Disease and Public Health, City University of Hong Kong, Kowloon, Hong Kong

**General comments:**

 The article was a questionnaire-based cross-sectional survey among undergraduate students (knowledge, attitude, and practices) in the mentioned study area on avian influenza. The report made it clear that the veterinary students learned more about this zoonotic disease than students of other faculties. The article preparation and structure are good, and they can be indexed. The manuscript has a clear layout, and the research findings fit the study objectives. Even though the author used clear English, there is enough scope to improvise sentence structures. Minor editing on references, tables, and English could improve the article.

**Abstract:**

 The abstract seems highly well. Methods, results, and conclusions are correlated to the study and understandable.

**Introduction:**

 Literature was clearly stated for publication, but some recent references would be good. A questionnaire-based survey and its importance should be a vital part of the introduction.

**Methods:**

 Methods are comprehensive, and I do believe they cover the idea. The use of “Few” in the methodology is a little improper. How many students from the other faculty already knew about the disease would correlate with their response to the questionnaire.

**Results:**

 The results could cover the objectives with the descriptive study, which could've done the regression analysis, but I think this could be enough for this report. However, the writing needs to be revised for more concise and precise. I would suggest one thing to consider - the author can make one pictorial presentation of the constructed tables.

**Discussion:**

 Excellently written already, add some new references, particularly previous 2/3 years. This part needs to be revised mainly keeping in mind that results should not be repeated.

Is the work clearly and accurately presented and does it cite the current literature?

Yes

If applicable, is the statistical analysis and its interpretation appropriate?

Yes

Are all the source data underlying the results available to ensure full reproducibility?

Yes

Is the study design appropriate and is the work technically sound?

Yes

Are the conclusions drawn adequately supported by the results?

Yes

Are sufficient details of methods and analysis provided to allow replication by others?

Yes

Reviewer Expertise:

Veterinary Epidemiology, Food Microbiology, Food Safety, One Health

I confirm that I have read this submission and believe that I have an appropriate level of expertise to confirm that it is of an acceptable scientific standard.

# Peer review: Reviewer response for version 1

**DOI:** 10.5256/f1000research.77920.r121510

**Published:** 2022-02-21

**Authors:** Sohail Ahmad

**Affiliations:** 1 Department of Department of Poultry Production, University of Veterinary and Animal Sciences, Lahore, Pakistan

First of all, thank you very much for giving me the opportunity to review this article, I have completed my evaluation and after reading this article carefully I am of the opinion that this manuscript should be considered for indexing. 

 The idea is very well-conceived and presented clearly, objectives are well organized and results are completely justifying the hypothesis. In general, this manuscript provides sound information and a good contribution in the field of Poultry Science (Poultry Diseases). I have provided some further comments and queries here.

 Thank you very much and please let me know if I can be of further assistance

Is the work clearly and accurately presented and does it cite the current literature?

Yes

If applicable, is the statistical analysis and its interpretation appropriate?

Yes

Are all the source data underlying the results available to ensure full reproducibility?

Yes

Is the study design appropriate and is the work technically sound?

Yes

Are the conclusions drawn adequately supported by the results?

Yes

Are sufficient details of methods and analysis provided to allow replication by others?

Yes

Reviewer Expertise:

Poultry Science

I confirm that I have read this submission and believe that I have an appropriate level of expertise to confirm that it is of an acceptable scientific standard.
